# Electrochemical and Colorimetric Nanosensors for Detection of Heavy Metal Ions: A Review

**DOI:** 10.3390/s23229080

**Published:** 2023-11-09

**Authors:** Sayo O. Fakayode, Charuksha Walgama, Vivian E. Fernand Narcisse, Cidya Grant

**Affiliations:** 1 Department of Chemistry, Physics and Astronomy, Georgia College and State University, Milledgeville, GA 31061, USA; 2 Department of Physical and Applied Sciences, University of Houston-Clear Lake, Houston, TX 77058, USA; walgama@uhcl.edu; 3 Department of Chemistry, Forensic Science and Oceanography, Palm Beach Atlantic University, West Palm Beach, FL 33401, USA; vivian_fernandnarcisse@pba.edu (V.E.F.N.); cidya_grant@pba.edu (C.G.)

**Keywords:** heavy metal ion detections, portable electrochemical nanosensors, colorimetric nanosensors, review

## Abstract

Human exposure to acute and chronic levels of heavy metal ions are linked with various health issues, including reduced children’s intelligence quotients, developmental challenges, cancers, hypertension, immune system compromises, cytotoxicity, oxidative cellular damage, and neurological disorders, among other health challenges. The potential environmental HMI contaminations, the biomagnification of heavy metal ions along food chains, and the associated risk factors of heavy metal ions on public health safety are a global concern of top priority. Hence, developing low-cost analytical protocols capable of rapid, selective, sensitive, and accurate detection of heavy metal ions in environmental samples and consumable products is of global public health interest. Conventional flame atomic absorption spectroscopy, graphite furnace atomic absorption spectroscopy, atomic emission spectroscopy, inductively coupled plasma–optical emission spectroscopy, inductively coupled plasma–mass spectroscopy, X-ray diffractometry, and X-ray fluorescence have been well-developed for HMIs and trace element analysis with excellent but varying degrees of sensitivity, selectivity, and accuracy. In addition to high instrumental running and maintenance costs and specialized personnel training, these instruments are not portable, limiting their practicality for on-demand, in situ, field study, or point-of-need HMI detection. Increases in the use of electrochemical and colorimetric techniques for heavy metal ion detections arise because of portable instrumentation, high sensitivity and selectivity, cost-effectiveness, small size requirements, rapidity, and visual detection of colorimetric nanosensors that facilitate on-demand, in situ, and field heavy metal ion detections. This review highlights the new approach to low-cost, rapid, selective, sensitive, and accurate detection of heavy metal ions in ecosystems (soil, water, air) and consumable products. Specifically, the review highlights low-cost, portable, and recent advances in smartphone-operated screen-printed electrodes (SPEs), plastic chip SPES, and carbon fiber paper-based nanosensors for environmental heavy metal ion detection. In addition, the review highlights recent advances in colorimetric nanosensors for heavy metal ion detection requirements. The review provides the advantages of electrochemical and optical nanosensors over the conventional methods of HMI analyses. The review further provides in-depth coverage of the detection of arsenic (As), cadmium (Cd), chromium (Cr), copper (Cu), mercury (Hg), manganese (Mn), nickel (Ni), lead (Pb), and zinc (Zn) ions in the ecosystem, with emphasis on environmental and biological samples. In addition, the review discusses the advantages and challenges of the current electrochemical and colorimetric nanosensors protocol for heavy metal ion detection. It provides insight into the future directions in the use of the electrochemical and colorimetric nanosensors protocol for heavy metal ion detection.

## 1. Introduction and Overview

The industrial revolution and radical technological advancement in the past decades increased the global population, urbanization, manufacturing and transportation of consumable goods, and access to health care. Technological advancement has also facilitated and improved standards of living, quality of human life, and life expectancy. Nonetheless, demand for the industrial revolution, technological advancement, and global population growth has come at a severe cost with unintended negative impacts on ecosystems and natural resources. For instance, unintended consequences of anthropogenic activity and industrial development have generated and liberated tons of environmental waste materials and toxic chemicals of concern, including toxic heavy metal ions, into the ecosystem (soil, water, and air). The influx of untreated industrial effluent, municipal wastes, agricultural and urban runoff into surface rivers, landfilled metal and electronic waste, automobiles, and mechanic shops continue to pose significant challenges in terms of environmental heavy metal ion contamination and ecological degradation. The aging of infrastructure, corrosion, the degradation of municipal water and sewage distribution piping systems, coal burning, construction, oil and mining, metallurgy, smelters, leather tanning, electroplating, inorganic dyes, batteries, petrochemicals, paints, agrochemicals, and the chemical fertilizers industry constitute primary sources of environmental heavy metal ion contamination [1,2,3,4,5,6,7,8]. Ecosystems, including humans, are exposed to heavy metal ion contaminations through direct point and non-pollution sources, occupational exposure, dietary intake of heavy metal-contaminated water or polluted food items, or inhalation of heavy metal from air particulates. 

Ecological and public health risk factors from the exposure to heavy metals is concerning because of the long residence time of heavy metals in the environment. Chronic and acute concentrations and heavy metal accumulation have been well documented and widely reported in soil and sediment, surface water, underground water, plants, food crops, fish, seafood, aquatic animals, and terrestrial animals [9,10,11,12,13,14,15,16,17,18,19,20,21,22,23,24,25,26,27,28,29,30,31,32,33,34,35,36,37]. Heavy metals are susceptible to bioaccumulation and biomagnification in plants, animals, and human organs via the food chain and trophic levels. Studies have also reported acute to chronic heavy metal ion toxic effects in human organs [10,11,12,13,14,15,16,17,18,19,20,21,22,23,24,25,26,27,28,29,30,31,32,33,34,35,36]. The exposure of animals and plants to heavy metal contamination and the resulting health risk factors, including oxidative stress, ecotoxicities, phytotoxicity, and physicochemical and biochemical changes on animals and plants, are concerning [38,39,40,41,42,43]. In addition, various health hazards, including reduced children’s intelligence quotients, developmental challenges, cancers, elevated blood pressure, immune system compromises, cytotoxicity, oxidative cellular damage, cardiovascular diseases, myocardial infarction, neurological disorders, and miscarriages and stillbirths, among other health challenges, have also been linked to elevated levels of heavy metals in humans [44,45,46,47,48,49,50,51,52,53,54,55,56,57,58,59,60,61,62]. The recent lead tap water crisis in Flint, Michigan, and the resulting health risk factors and social and post-traumatic stress disorders are a wake-up call for being ready for potentially widespread heavy metal contaminations [63,64,65,66,67,68,69,70]. Developing effective environmental heavy metal ion detections is paramount to ensuring public health safety and global homeland security.

### Conventional Heavy Metal Ion Analysis and Trace Element Detection Methods

**Figure 1:**
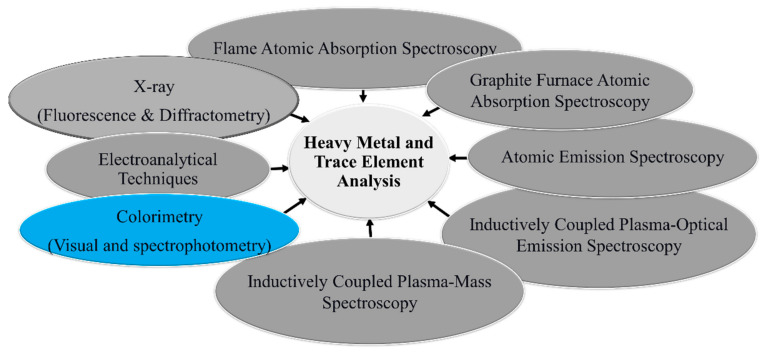
Conventional instrumental methods of heavy metal ion and trace element analysis in environmental samples.

Notable progress has been made in developing capable analytical protocols for detecting, analyzing, and screening heavy metal ions in environmental samples (Figure 1). For instance, conventional flame atomic absorption spectroscopy (FAAS), graphite furnace atomic absorption spectroscopy (GF-AAS), atomic emission spectroscopy (AES), inductively coupled plasma–optical emission spectroscopy (ICP-OES), inductively coupled plasma–mass spectroscopy (ICP-MS), X-ray diffractometry, and X-ray fluorescence [71,72,73,74,75,76,77,78,79,80,81,82,83,84] have been well-developed for heavy metal and trace elements. Nonetheless, some techniques have significant drawbacks and challenges that limit their practical applications. For example, FAAS requires a large sample size, flammable fuels, and a relatively expensive cathode lamp for each element (though a multi-element cathode lamp is available, it suffers from element interference). Background, spectra, and chemical ionization interferences are problematic in FAAS. The oxidation of carbon graphite in GF-AAS constitutes a challenge in GF-AAS. ICP-OES and ICP-MS instruments require ultra-pure argon gas. X-ray diffractometry is expensive and less sensitive for metal ion analysis at ultra-trace levels.

In addition to high instrumental running and maintenance costs and specialized personnel training, these instruments are not portable, limiting their practicality for on-demand, in situ, field study, or point-of-need heavy metal ion detection. Electrochemical and colorimetric nanosensors are viable alternative strategies that address some of the challenges of the available HMI detection methods. Toward this effort, various electrochemical nanosensors have generated significant interest in detecting heavy metal ions in recent years [85]. Increases in the use of electrochemical techniques for heavy metal ion detections arise because of high sensitivity and selectivity, cost-effectiveness, a small size requirement, and the rapidity of electrochemical methods. For instance, electrochemical sensors also have portable instrumentation and are sometimes disposable, facilitating on-demand, in situ, and field heavy metal ion detections. Hu et al. 2023 have comprehensively reviewed the advances and advantages of portable heavy metal analysis sensors [85].

In addition, electrochemical sensors and colorimetric nanosensors have been developed, allowing rapid screening and visual detection for point-of-use, in situ, and field heavy metal ion detections. In a review article, Ullah et al. 2018 recently emphasized innovation and advancements of nanomaterial-based optical sensors for heavy metal analysis [86]. This review highlights the new approach to low-cost electrochemical and colorimetric nanosensors for fast, selective, sensitive, and accurate detection of HMIs in ecosystems (soil, water, and air) and biological samples. Specifically, this review highlights low-cost portable electrochemical nanosensors and recent advances in smartphone-operated screen-printed electrodes (SPEs), plastic chip SPES, and carbon fiber paper-based and microfluidic-based nanosensors for selective environmental heavy metal ion detection. This review also provides up-to-date advances in optical sensors for the fast detection and analysis of heavy metal ions. In addition, this review provides in-depth coverage of the detection of Arsenic(III) and Arsenic(IV), Cr(III) and Cr(IV), Hg(I) and Hg(II), Pb(II) and Pb(IV), Manganese (II), Fe(II), and Fe(III) ions in environmental and biological samples and consumable products. Moreover, the review discusses the advantages and shortcomings of the current electrochemical and colorimetric nanosensors protocol for heavy metal ion detection and future directions for heavy metal ion detection.

## 2. Portable Electrochemical Nanosensors for the Detection of Heavy Metal Ions in Environmental Samples

Heavy metal pollution is a significant environmental concern due to its potential to harm ecosystems, human health, and aquatic life. Heavy metals become pollutants when they enter water bodies at elevated concentrations, often due to industrial processes, urban runoff, and anthropogenic activities. Some common heavy metals of concern in wastewater include lead, mercury, cadmium, chromium, arsenic, and nickel. Continuous water quality monitoring and research into the sources and effects of heavy metal pollution are essential for developing effective mitigation strategies. Among various analytical techniques, electrochemical detection is a powerful and widely used method for quantifying and monitoring heavy metals in different environmental samples, including water, soil, and air [87,88]. In this regard, portable and disposable electrochemical sensors have generated considerable attention because of their ease of use, cost-effectiveness, and suitability for on-site monitoring [89,90,91,92,93]. These sensors are designed to be user-friendly, providing rapid and reliable measurements without the need for extensive sample preparation or sophisticated equipment. Their sensitivity and selectivity towards heavy metal pollutants are critical in method development. Nanomaterials play a fundamental and crucial role in the fabrication process of electrochemical sensors. Nanomaterials offer stable support structures and highly active sites for functionalization, making them excellent candidates for improving the selectivity and sensitivity of electrodes in heavy metal detection [92].

In recent years, there has been a growing interest in leveraging nanomaterials to enhance the effectiveness of electrode surfaces in detecting heavy metals. Modified electrodes incorporating nanomaterials have proven decisive in electroanalytical methods for identifying a wide range of heavy metals. Such nanomaterials include metal nanoparticles, metal oxides, graphene-based materials, carbon nanotubes, and metal–organic frameworks (MOFs). Standard nanomaterial fabrication techniques on disposable electrodes include drop casting, dip coating, spin coating, electrochemical deposition, direct growth, and screen printing [93]. Table 1 summarizes the latest studies that have employed various nanostructure architectures in conjunction with screen-printed electrodes (SPE) for the portable detection of heavy metal pollutants in environmental samples.

Huang and coworkers [94] have demonstrated that phosphorus-doped biochar–attapulgite/bismuth film electrodes decorated with magnetic Fe_3_O_4_ nanoparticles (MBA-BiFE) can be utilized to detect Cd(II), Pb(II), and Hg(II) with limits of detection of 0.036 nM, 0.003 nM, and 0.011 nM, respectively. They showed that a machine learning model based on an artificial neural network (ANN) can perform multi-metal analysis using the data generated from a portable wireless smart sensor, connected to the modified screen-printed electrode [94]. Similarly, core-shell Fe_3_O_4_@Au nanoparticles anchored with cysteamine have been used to prepare a composite with thymine acetic acid (Fe_3_O_4_@Au/CA/T-COOH) for Hg(II) detection in the range of 1–200 µg/L in wastewater samples [95]. Moreover, silver nanowires and butterfly-shaped silver nanoparticles have been successfully employed to detect Cd(II), Pb(II), Cu(II), and Hg(II) in ppb levels using disposable SPEs [96,97]. Among carbonaceous nanomaterials, carbon nanotubes and graphene are widely employed to modify screen-printed electrodes. Hajzus et al. [98] investigated a sensitive platform for the selective voltammetric measurement of CdCl_2_, CuSO_4_, HgCl_2_, and PbCl_2_ in seawater based on epitaxial graphene-modified SiC paper. They implemented machine learning models to accurately identify heavy metal types based on cyclic square wave voltammograms. In another study, Bao et al. [99] constructed an SPE modified with chitosan/PANi–Binanoparticle@graphene oxide multi-walled carbon nanotubes (CS/PANi–Bi NP@GO–MWCNT) for the rapid detection of Cu(II) and Hg(II) ions. Their portable detection platform comprises an in situ signal analysis circuit, a Bluetooth chip, a photocured 3D-printed shell, and an electrode sleeve interface. This portable electrochemical sensor was tested for Hg (II) and Cu (II) with detection limits of 10 ppb and 0.998 ppm, respectively, as given in Table 1.

Metal–organic frameworks (MOFs) are a novel category of nanoporous materials that demonstrate efficacy as a highly effective platform for the electrochemical sensing of heavy metals. MOFs can be designed with specific pore sizes and functional groups tailored to capture and bind to heavy metal ions selectively. Tan et al. [100] developed a novel hybrid material featuring a hetero-shelled hollow structure composed of metal–organic framework (MOF) components, denoted as HCZ@UN. This involved utilizing hollow carbonized ZIF-8 (HCZ) as a substrate for the growth of UiO-66(Zr)–NH_2_ (UN) on an SPE for the efficient detection of Pb (II) ions in tap water samples in the range of 0.100–500 nM. Qi and coworkers designed an electrochemical sensor for Cd (II) detection using a complex of carbon fiber paper (CFP), CoMOF, AuNPs, and glutathione as the conductive substrate (CFP/CoMOF/AuNPs/GSH). They achieved Cd (II) detection as low as 1 nM [101]. In a related study, Wang et al. employed a covalent organic framework prepared with the condensation of 2,5-diamino-1,4-phenyldicarboxylic acid (DATA), and 2,4,6-triformylphloroglucinol (TP). Here, the uniformly distributed -COOH and NH groups on the pore’s wall were utilized as heavy metal ion adsorption sites. The electrochemical sensor could detect Hg(II), Cu(II), Pb(II), and Cd(II) simultaneously, and the limit of detection was at sub-nanomolar levels [102] (See Table 1).

In addition to the nanostructures mentioned above, various other materials are also utilized to modify SPEs. These materials include chemically functionalized isoporous [103] and mesoporous [104] silicon membranes, ion-imprinted polymer films [105], and electrodeposited bismuth films [106,107]. These modifications have been employed for the sensitive and selective detection of several heavy metal ions such as Cd(II), Pb(II), Cu(II), Hg(II), Zn(II), and As(III) in environmental samples, as detailed in Table 1.

### 2.1. Portable Electrochemical Nanosensors for the Detection of Heavy Metal Ions in Biological Samples

Diagnosis of heavy metals in humans typically involves blood or urine laboratory tests to measure the concentrations of heavy metals in the body using inductively coupled plasma–mass spectrometry (ICP-MS). Table 2 indicates the reference levels based on the CDC guidelines and heavy metal screening blood and urine test catalogs of Mayo Clinic Laboratories.

**Table 1:** Summary of recent reports on the detection of heavy metals in environmental samples using nanostructure-modified electrodes.

Electrode Modification	Electrochemical Detection Method	Portability	Metal Ions	Linearity	LOD	Sample	Ref.
Phosphorus-doped biochar–attapulgite/bismuth film electrode decorated with magnetic Fe_3_O_4_ nanoparticles (MBA-BiFE)	SWASV	Smartphone-operated SPE	Cd(II) Pb(II) Hg(II)	0.1 nM–5 μM, 0.01 nM–7 μM, 0.1 nM–3 μM	0.036 nM 0.003 nM 0.011 nM	Tap water Lake water	[94]
Thymine acetic acid anchored with cysteamine-conjugated core-shell Fe_3_O_4_@Au nanoparticles (Fe_3_O_4_@Au/ CA/T-COOH)	DPASV	SPE/plastic chip sample holder	Hg(II)	1–200 μg/L and 200–2200 μg/L	0.5 μg/L	Wastewater	[95]
Butterfly-shaped silver nanostructure (AgNS)	DPASV	SPE	Cd(II) Pb(II) Cu(II) Hg(II)	5–300 ppb 5–300 ppb 50–500 ppb 5–100 ppb	0.4 ppb 2.5 ppb 7.3 ppb 0.7 ppb	Tap water Rainwater Lake water	[96]
Silver nanowires, hydroxymethyl propyl cellulose, chitosan, and urease (AgNWs/HPMC/CS/Urease)	CV	SPE	Hg(II)	5–25 μM	3.94 μM	Drinking water	[97]
Epitaxial Graphene on SiC	CSWASV	Portable in-house built potentiostat	CdCl_2_ CuSO_4_ HgCl_2_ PbCl_2_	Spiked samples 100–3000 ppb	-	Sea water	[98]
Chitosan/PANi–Bi nanoparticle@graphene oxide multi-walled carbon nanotubes (CS/PANi–Bi NP@GO–MWCNT)	DPV	Portable device with an in situ signal analysis circuit, a Bluetooth chip, a photocured 3D-printed shell, and an electrode sleeve interface	Hg(II) Cu(II)		10 ppb 0.998 ppm	Tap water	[99]
Zirconium-based MOF material, UiO-66(Zr)–NH_2_	DPASV	SPE	Pb(II)	0.100–500 nM	0.0492 ± 0.00523 nM	Tap water	[100]
Carbon fiber paper, CoMOF, AuNPs, and glutathione (CFP/CoMOF/AuNPs/GSH)	SWV	Carbon fiber paper electrode	Cd(II)	0.001–1 μm	1.0 nM	Lake water River water	[101]
Covalent organic framework (COFDATA-TP)	SWASV	SPE	Hg(II) Cu(II) Pb(II) Cd(II)	0.0085–8.00 μM 0.015–8.00 μM 0.0056–8.00 μM 0.0069–8.00 μM	2.80 nM 5.01 nM 1.83 nM 2.91 nM	River water	[102]
Silica isoporous membrane (SIM)	SWASV	SPE	Cd(II) Pb(II) Cu(II) Hg(II)	0.2–20.0 µM 0.01–10.0 µM 0.2–20.0 µM 0.01–10.0 µM	9.3 nM 1.1 nM 16.2 nM 1.4 nM	Soil	[103]
Chemically decorated mesoporous silica (SBA-15 and MCM-41) with L-cysteine (L-cys).	SWV	-	SBA-15 Cd(II) Pb(II) MCM-41 Cd(II) Pb(II)	5–80 μg/L 10–80 μg/L 5–80 μg/L 10–80 μg/L	0.22 μg/L 0.36 μg/L 0.23 μg/L 0.76 μg/L	Tap water Lake water	[104]
Ion-imprinted polymer film (IIP)	CV	SPE	Cd(II)	10–1200 nM	1.71 nM	Drinking water Tap water Marine water	[105]
Screen–printed gold working electrode with electroplated bismuth film (Bi/SPAuE)	SWASV	SPE	Pb(II) Cd(II) Zn(II)	10–120 µg/L	0.04 μg/L 0.02 μg/L 0.23 μg/L	Industrial wastewater	[106]
Hg/Bi-plated glassy carbon electrode	SWASV LSASV	-	Cd(II) Pb(II) As(III)	-	0.03 μg/L 0.05 μg/L 0.15 μg/L	Tap water Mountain spring water River water	[107]

DPASV—differential pulse anodic stripping voltammetry; SWASV—square wave anodic stripping voltammetry; CV—cyclic voltammetry; SWASV—square wave anodic stripping voltammetry; SWV—square wave voltammetry; DPV—differential pulse voltammetry; LSASV—linear sweep anodic stripping voltammetry.

**Table 2:** Reference range of heavy metals in human biofluids.

Metal	Normal Values *	
	Blood	Urine
As	<13 ng/mL (all ages)	0–17 years: Not established > or =18 years: <24 g/g creatinine
Pb	0–5 years: <3.5 g/dL > or =6 years: <5.0 g/dL Critical values Pediatrics (< or =15 years): > or =20.0 g/dL Adults (> or =16 years): > or =70.0 g/dL	0–17 years: Not established > or =18 years: <0.6 g/g creatinine
Cd	<5.0 ng/mL (all ages)	0–17 years: Not established > or =18 years: <2 g/g creatinine
Hg	<10 ng/mL (all ages)	0–17 years: Not established > or =18 years: <2 g/g creatinine

* Reference levels are reported based on the CDC guidelines and Heavy Metals Screen with Demographics, Blood (HMDB) test and the Heavy Metal/Creatinine Ratio with Reflex, Random, Urine (HMUCR) test provided by Mayo Clinic Laboratories as of 09/2023 [108,109].

Given that blood is an intricately complex biological fluid, there have been recent advancements in the development of portable electrochemical sensor platforms designed for the non-invasive detection of heavy metals in bodily fluids such as urine, saliva, and sweat [110,111,112]. This review section focuses on recent developments in portable electrochemical devices capable of detecting heavy metals within clinically relevant concentration ranges.

**Figure 2:**
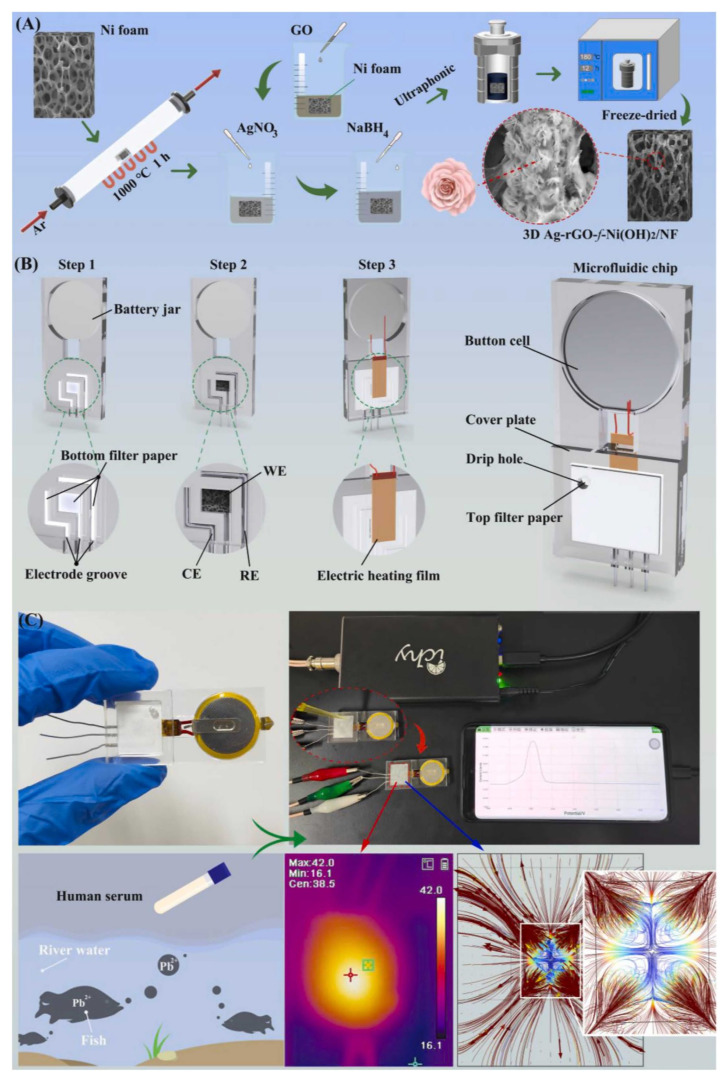
(**A**) The preparation of 3D Ag-rGO-f-Ni(OH)_2_/NF composites, (**B**) The manufacturing process of the microfluidic device, (**C**) The 3D Ag-rGO-f-Ni(OH)_2_/NF microfluidic sensor for electrochemical sensing Pb^2+^ Reproduced from Ref. [112] with permission from Elsevier.

Ma and their research team have designed a microfluidic electrochemical sensing chip that relies on a smartphone-based electrochemical workstation to detect Pb^2+^ in human serum (see Figure 2) [112]. To enhance the surface area and conductivity, they have harnessed a nanocomposite of silver nanoparticles, reduced graphene oxide, and nickel hydroxide on a nickel form (Ag-rGO-f-Ni(OH)_2_/NF) as the working electrode. Furthermore, they have shown that incorporating a thermocapillary convection process within the microfluidic platform promotes electrolyte flow and expedites electron transfer, reducing assay times and amplifying electrochemical signals. This innovative device could generate differential pulse voltammetry (DPV) signals for Pb(II) within the 0.01–2100 µg/L concentration range.

Similarly, Wang and colleagues have introduced a microfluidic paper-based analytical device (μPAD) capable of isolating proteins and detecting lead ions in urine samples [113]. Proteins are well-known for fouling electrodes, posing a challenge for the direct electrochemical detection of heavy metals in urine. To address this issue, the authors have modified the sample zone of the paper device with (NH_4_)_2_SO_4_ to precipitate urinary proteins through a salting-out effect upstream of the detection zone. This portable paper sensor exhibits a linear range of 10–500 μg/L with a detection limit of 9 μg/L for detecting Pb(II) in urine, utilizing anodic stripping voltammetry (ASV).

Magnetic sorbents offer a unique combination of impressive sorption capacity while being conveniently manipulated by an external magnetic field, eliminating the need for labor-intensive filtration or centrifugation processes during phase separation. This dual advantage reduces the overall operation time and enhances the portability and feasibility of on-site extractions, making the procedure more accessible and efficient. In a distinct study, Fernández and their team employed magnetic dispersive solid-phase extraction (MDSPE) in conjunction with electrochemical detection, utilizing a screen-printed carbon electrode to determine the presence of Pb(II). In addition to lead, similar electrochemical approaches have been successfully integrated into microfluidic portable platforms for the detection of cadmium [114,115,116], mercury [117], and zinc [118].

### 2.2. Portable Electrochemical Nanosensors for the Detection of Heavy Metal Ions in Food Samples

Heavy metal food contamination is a significant and concerning issue with far-reaching implications for public health, agriculture, and the environment [119,120]. Unfortunately, due to escalating environmental and industrial pollution levels, heavy metals have become pervasive in everyday food items, including vegetables, fruits, meat, marine food, and water sources. Therefore, it is essential to institute measures to detect and continually monitor heavy metal levels in our food supply. In this context, this section presents a summary of the most recent articles published on the detection of heavy metals in food. As discussed in the previous sections, using paper-based SPEs with nanostructure modifications on the transducer surface enhances the portability and sensitivity of the electrochemical detection platform [121,122]. Furthermore, a distinct sample preparation method must be employed when dealing with actual food samples to extract heavy metals into an acidic matrix after digestion or an ashing process.

**Figure 3:**
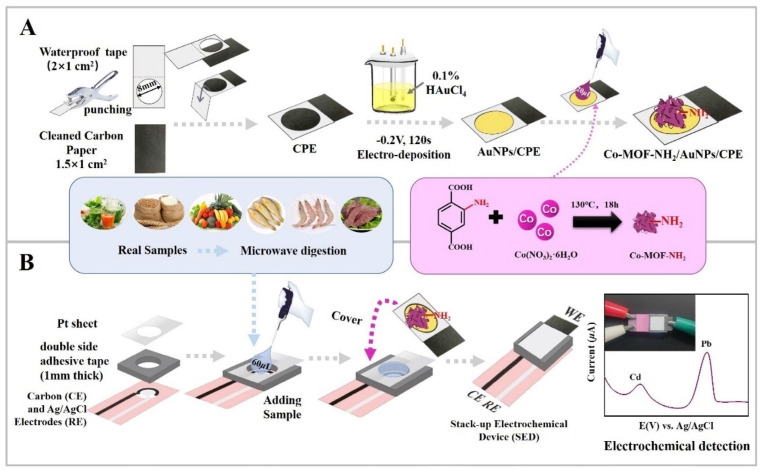
(**A**) The preparation of Co-MOF-NH_2_/AuNPs/CPE and (**B**) analytical procedure on stack-up electrochemical device. Reproduced from Ref. [123] with permission from Elsevier.

Recently, Pang et al. [123] developed a stack-up electrochemical device modified with an amino-functionalized cobalt-based metal–organic framework and gold nanoparticles (Co-MOF-NH_2_/AuNPs/CPE) to detect heavy metals in various food samples. Their method enabled the simultaneous detection of Pb(II) and Cd(II) with detection limits of 7.0 × 10^−2^ and 1.1 × 10^−2^ ng/mL, respectively, in natural food samples such as drinking water, juice, tea, grains, fruits, vegetables, liver, and aquatic products. In the sample preparation process, solid samples were initially crushed using a tissue shredder and then digested using concentrated nitric acid and a 30% hydrogen peroxide solution. Subsequently, the extracts were decomposed using a microwave and heated on a graphite digestion apparatus until they were mixed with an acetate buffer (pH 5.0) for the final analysis [123] (See Figure 3).

Pungjunun and colleagues have introduced a sensor design featuring a bismuth nanoparticle-modified screen-printed graphene electrode (BiNP/SPGE) integrated into a paper-based analytical device. This setup allows for the simultaneous determination of Sn(II) and Pb(II) while incorporating a portable potentiostat for enhanced mobility and convenience. Under optimal conditions, the linear range for both metals spans from 10 to 250 ng/mL, with a calculated limit of detection values of 0.26 and 0.44 ng/mL for Sn(II) and Pb(II), respectively. This device has been utilized to analyze the above heavy metal ions in canned food samples (mushrooms and bamboo shoots). Solid samples were ground into a fine powder using a blender and digested with a 2% *v*/*v* HNO_3_ solution. Then, the pH was adjusted to pH 7 using NaOH solution and diluted with oxalic acid and cetyltrimethylammonium bromide (CTAB) for electrochemical analysis.

Rice is a staple food globally, especially in Asia, Africa, and the Middle East. Rice is prone to take up and bioaccumulate heavy metals, especially Pb, Cd, As, and Hg, from contaminated soil or irrigated agricultural water. The intake of heavy metal contaminated rice can pose health risks to consumers, as these heavy metals are toxic with negative health implications, including organ damage and cancer. Jiang et al. recently developed a smartphone-based electrochemical cell to evaluate the toxicity of Cd(II), Pb(II), and Hg(II) ions on Hep G2 cells as an indirect measurement of heavy metals in the analyte sample [124]. Here, the sensor was fabricated with reduced graphene oxide (RGO)/molybdenum sulfide (MoS_2_) composites to significantly improve the biological adaptability for immobilizing Hep G2 cells. Differential pulse voltammetry (DPV) was employed to measure the electrical signals induced by the toxicity of heavy metal ions. The IC_50_ values for Cd(II), Pb (II), and Hg (II) were calculated as 49.83 μM, 36.94 μM, and 733.90 μM, respectively, by the electrochemical method. They utilized those cytotoxicity curves (the curve between heavy metal concentration and cell inhibition rate) to quantify levels of heavy metals in spiked rice samples after a wet digestion process. In another study, a glassy carbon electrode modified with silver nanoparticles (AgNP), bismuth nanoparticles (BiNP), multiwalled carbon nanotubes (MWCNT), and Nafion was utilized to detect Cd(II) and Pb(II) with the LODs of 25.12 ppb and 20.55 ppb, respectively. The rice samples were analyzed after an ashing and acid digestion process [125].

Voltammetry, impedimetry, potentiometry, conductometry, and amperometry represent the primary techniques employed in the electrochemical detection of heavy metals in all the studies mentioned above [126]. Various methodologies are utilized within the realm of voltammetry, including cyclic voltammetry, linear sweep voltammetry, differential pulse voltammetry, and square wave voltammetry. Additionally, stripping voltammetry comprises three fundamental variants: anodic stripping voltammetry (ASV), cathodic stripping voltammetry (CSV), and adsorptive stripping voltammetry (AdSV). The essence of stripping analysis lies in a two-step process: an initial pre-concentration step on the working electrode (reduction), followed by a subsequent step that removes the accumulated heavy metal ions from the electrode’s surface (oxidation) through a Faradaic reaction, thereby returning the heavy metal ions into the solution. This final process generates a current signal proportional to the solution’s heavy metal concentration.

## 3. Colorimetric (UV-Visible) Nanosensors for Heavy Metal Ion Detections

**Figure 4:**
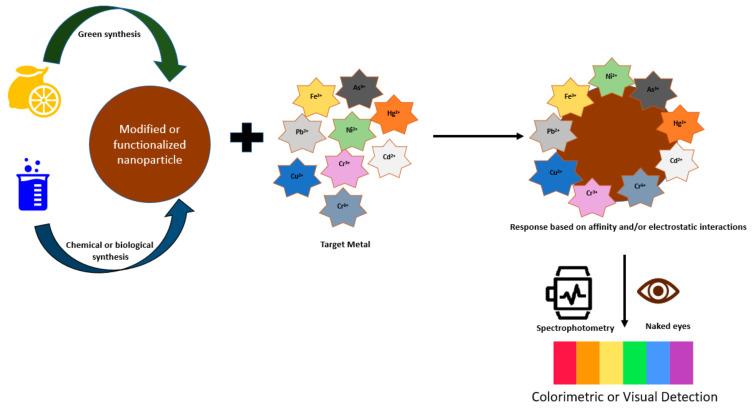
Scheme of colorimetric-based nanosensors for the detection of heavy metals.

Colorimetric nanosensors can be categorized, according to their route/manner of synthesis, into (1) green synthesis nanomaterials and (2) chemical or biological synthesis nanomaterials [127]. The overall principle of colorimetric nanosensors is based on the binding and affinity interaction between nanosensors and metal ions, causing a change in absorbance (Figure 4). The development of colorimetric nanosensors is strongly associated with the type of fabrication material. The mode of action for colorimetric detection can be attributed to (1) localized surface plasmon resonance (LSPR) phenomenon and (2) nanozyme or nanozyme-like properties of the material. LSPR-based colorimetric sensors are usually fabricated from metal nanoparticles (e.g., gold, silver) since they each have a specific absorbance band, giving them a selective response to heavy metals. The principle of LSPR-based colorimetric sensors relates to a color change of the metal nanoparticle via an aggregation or etching process, stemming from the characteristic absorption band. For the synthesis of nanozyme-assisted colorimetric sensors, nanoscale materials with catalytic properties are used that can detect low concentrations of heavy metal ions. The catalytic activity of these nanozymes can be stimulated or inhibited when metal ions interact or absorb on the nanozyme surface [128,129]. During these chemical reactions, a color change is produced. Table 3 summarizes the various types of colorimetric sensors reviewed in this section.

### 3.1. Colorimetric Nanosensors for Detecting Heavy Metal Ions in Environmental Samples

#### 3.1.1. Detection of Cu^2+^ Ions

Copper is a heavy metal with a significant role in environmental pollution due to its abundant presence in aquatic environments (e.g., marine and freshwater habitats) and industrial runoffs. It is toxic at high concentrations to all living species. Ghasemi and Mohammadi [130] developed a novel thiazolylazopyrimidine-functionalized TiO_2_ nanosensor (TiO_2_-TAP) to detect Cu^2+^ in aquatic samples. They synthesized thiazolylazopyrimidine (TAP), an azo ligand that contained N, S, and O functional groups as binding sites. The TAP ligand has the azo chromophore (N=N), which can generate a color and form a stable complex with Cu^2+^ grounded on the charge-transfer transduction process during detection. The ligand was activated with epoxy, a surface modifier, and reacted with titanium dioxide nanoparticles to form TiO_2_-TAP NPs. The nanosensors were assessed by various characterization techniques. An aqueous solution of TiO_2_-TAP NPs was then used to detect Cu^2+^ ions by examining its ability to adsorb these ions from the aqueous media. Due to surface complexation, the nanosensor solution turned from yellow to red within a few seconds upon adsorption of Cu^2+^ ions. The maximum absorbance of the resulting complex solution was 536 nm. In addition, the linear range for detecting Cu^2+^ ions in aqueous media was between 0.01 and 12.5 µM, and the limit of detection (LOD) was 2.51 nM. The optimum adsorption of Cu^2+^ ions to TiO_2_-TAP NPs from tap water, seawater, and well water occurred at pH 5 and was selective. Moreover, the sensor’s response time was short, with a high adsorption efficiency towards Cu^2+^ (after 30 min, 93% of the copper ions were adsorbed). Furthermore, the authors report that the design and fabrication of this sensitive nanosensor were straightforward and inexpensive [130].

#### 3.1.2. Detection of Cr^3+^ and Cu^2+^ Ions

Wang and coworkers [131] developed multi-functional iodide-assisted silver nanoplates by coating the surface with citrate and iodide ions for the selective and sensitive colorimetric detection of chromium (III) and copper (II) ions in tap and lake water samples, respectively. The detection of Cr^3+^ by citrate-capped silver nanoplates was based on the aggregation of silver nanoplates due to the affinity of Cr^3+^ ions for the carboxylate groups of citrates, which cause the solution’s color to change from deep yellow to purple and finally to colorless. The synthesized colloidal silver nanoplates were evaluated by several characterization techniques. When Cr^3+^ ions were added to the citrate-functionalized silver nanoplates, the nanosensors’ hydrodynamic diameter increased from ~35 to ~379 nm. Furthermore, upon adsorption of Cr^3+^ to the surface of the silver nanoplates, the zeta potential value decreased as the Cr^3+^ concentration increased, which in turn triggered aggregation. The authors also found that the Cr^3+^ ions formed a coordination complex with the citrate-capped silver nanoplates, thereby neutralizing the surface charge. This increased the dispersion force between the Cr^3+^ ions and the nanosensor, causing aggregation. The silver nanoplates had a maximum absorbance of 390 nm. However, as Cr^3+^ ions were added, this peak decreased while a new absorption peak at 520 nm arose due to silver aggregation. At the same time, the authors noticed a color change (deep yellow to purple) in the solution. The linear range to detect Cr^3+^ based on A_390_/A_520_ was between 25 and 400 nM, and the LOD was calculated to be 8.0 nM. The colorimetric detection of Cu^2+^ by iodide-assisted silver nanoplates was based on fusion/oxidation etching of the silver nanoplates, causing the solution to change from deep yellow to colorless as the Cu^2+^ concentration increased. The morphology of the fused iodide silver nanoplates was assessed using TEM. When Cu^2+^ ions were added to the iodide-assisted silver nanoplates, the nanosensors’ hydrodynamic diameter increased from 68 to 133 nm. The zeta potential also increased, indicating that the surface charge of the silver nanoplates increased due to the fusion procedure and oxidation etching. The detection mechanism of Cu^2+^ was based on the adsorption of I^−^ onto the surface of the silver nanoplates and then the fusion/oxidation etching process, which was as follows: Cu^2+^ oxidizes I^−^ to I_2_ through the intermediate product CuI. Then, the formed I_2_ oxidizes silver on the silver nanosensors to AgI, changing from deep yellow to colorless as the Cu^2+^ concentration increases. The linear range for the detection of Cu^2+^ was measured at A_390_ and was between 0.3 and 10 µM, and the LOD was 0.27 µM. The researchers also indicated that the design of the iodide-assisted silver nanoplates was easy and fast [131].

#### 3.1.3. Detection of Fe^3+^, Cu^2+^, and Cr^6+^ Ions

In another study, Augen et al. [132] used green algae to synthesize Ag/AgCl nanoparticles (NPs) for colorimetric detection of Fe^3+^, Cu^2+^, and Cr^6+^ ions. Seaweed extract was used as a stabilizing and reducing agent as it contains an abundance of biomolecules (e.g., polysaccharides, proteins, lipids, polyphenols, and carotenoids). Moreover, green algae is known to be a source of Cl^−^ ions. During the synthesis of Ag/AgCl, the seaweed extract was combined with an AgNO_3_ solution (1:9, *v*/*v*) at 95 °C, which led to the formation of AgCl. Subsequently, the AgCl was stabilized by the organic compounds in the green extract. At the same time, some other Ag^+^ ions were reduced to Ag^0^ by reducing biomolecules (e.g., chlorophyll and phenols) in the seaweed extract. The precipitation of AgCl occurred at a high temperature, which increased its solubility, leading to its decomposition into its ions. In turn, these silver ions were also reduced to metallic silver. This change in equilibrium led to the formation of Ag@AgCl NPs. These green nanoparticles were evaluated using several characterization techniques. The Ag@AgCl NPs were spherical and ranged between 4 and 10 nm. The synthesized nanosensors were dark brown and had a maximum absorbance peak between 400 and 450 nm due to surface plasmon resonance (SPR) formation. The Ag@AgCl NPs had negatively charged surfaces because of the functional groups (amino, carboxyl, and hydroxyl) in the green algae, which led to the aggregation of the nanoparticles upon the addition of the metal ions. Upon adding Fe^3+^ and Cu^2+^ metal ion solutions to the nanosensors, the characteristic SPR band of Ag@AgCl NPs disappeared. Furthermore, the metal ion absorbance peak was slightly around the Ag@AgCl NPs absorbance peak with a shift toward the blue.

However, for Cr^6+^ metal ions, the authors noticed a decrease in the SPR peak of Ag@AgCl NPs with a redshift. In addition, a peak at 390 nm appeared for Cr^6+^ ions. Results showed that these Ag@AgCl NPs could detect the metal ions by forming different colors. Solutions of Ag@AgCl NPs turned in the presence of Fe^3+^, Cu^2+^, and Cr^6+^ ions from dark brown to light brown, white, and orange, respectively. The color intensity changes and the amount of aggregation of the nanoparticles were found to be dependent on the metal ions’ concentration. The LOD values of the metal ions Fe^3+^, Cu^2+^, and Cr^6+^ were 1.69, 3.18, and 5.05 ppb, respectively. The linear range for each metal ion was reported to be between 0 and 100 ppb. This eco-friendly synthesis method for these biogenic Ag@AgCl NPs was found to be inexpensive. Moreover, these nanosensors were highly sensitive for the concurrent colorimetric detection of Fe^3+^, Cu^2+^, and Cr^6+^ ions in wastewater [132].

#### 3.1.4. Detection of Pb^2+^ Ions

Li et al. [133] developed a plasma-based system for the instant and continuous green synthesis of glucose-functionalized gold nanoparticle (G-AuNPs) sensors for the colorimetric detection of Pb^2+^ ions. The authors utilized a one-pot microplasma system using plasma electrons as reducing agents to add Pb^2+^ functionalized groups (e.g., hydroxyl and carboxyl) from glucose to the surface of AuNPs, forming G-AuNPs. Thus, in the presence of Pb^2+^ ions, a G-AuNPs/Pb^2+^ complex was produced, causing a shift in the SPR and making colorimetric detection of Pb^2+^ ions possible. They also developed an advanced microfluidic plasma system for the continuous synthesis of G-AuNPs and simultaneous colorimetric detection of lead (II) ions within half a minute. The prepared functionalized gold nanosensors were evaluated using various characterization techniques. The absorption spectrum of the G-AuNPs showed an SPR peak at 530 nm, and the color of this nanosensor solution was claret-red. When aqueous Pb^2+^ ions were added to the G-AuNPs, an additional absorption peak appeared at 767 nm, while the one at 530 nm significantly decreased. At the same time, the color of the G-AuNPs changed to gray, indicating the formation of a G-AuNPs/Pb^2+^ complex as nanoparticle aggregation occurred. Results showed that Pb^2+^ ions form a coordination complex with the hydroxyl and carboxyl groups on the G-AuNPs surface. The nanosensor was highly sensitive and selective toward the in situ detection of Pb^2+^ ions. The LOD was 1.07 µM, and the linear range was between 10 and 80 µM [133].

#### 3.1.5. Detection of Hg^2+^ Ions

Tian and coworkers [134] developed *L*-cysteine functionalized graphene-oxide (CGO) nanosheets as nanosensors for the colorimetric detection of mercury ions (Hg ^2+^) in water samples. This metal-free chemical sensor was greenly synthesized at room temperature. The colorimetric detection of trace Hg^2+^ was based on the presence of sulfur and oxygen groups on the CGO that served as active sites for binding Hg^2+^. The interaction of the CGO, colorimetric substrate 3,3′,5,5′- tetramethylbenzidine (TMB), and Hg^2+^ was investigated and it was revealed that competitive adsorption existed between Hg^2+^ ions and TMB over the CGO, with the Hg^2+^ ions hindering the TMB from binding on the CGO. This facilitated the oxidation of TMB by peroxide, producing more colored oxidation products (darker blue), from which the colorimetric sensing of Hg^2+^ could be reached with good detection. A high efficiency and sensitive response to Hg^2+^ was demonstrated, with a detectable range of 0–200 µgL^−1^ and a detection limit of 7.6 µgL^−1^. This CGO-based colorimetric sensor also offered high selectivity for the target Hg^2+^ ions, with no detectible disturbance from other co-existing metal ions [134].

In a related study, graphene oxide (GO) stabilized silver nanoparticles (AgNPs) were created for the colorimetric detection of trace Hg^2+^ in environmental water samples [135]. Sugar beet bagasse, an agro-industrial waste product, was carbonized by combustion, and the recovered graphite powder was used to synthesize graphene oxide. The graphene oxide was then applied as a stabilizer of the silver nanoparticles. The Ag-GO nanoparticles (AgNPs) were prepared by an in situ reduction reaction of Ag^+^, which resulted in a yellow solution (λ_max_ 400 nm) of AgNPs. The sensor was then used to detect trace Hg^2+^. Here, the mechanism was based on an amalgam reaction between AgNPs and Hg^2+^ and resulted in an observed color change of the sensor solution from yellow to colorless. Their produced sensor exhibited practical application potential, with good sensitivity and selectivity to Hg^2+^ in the presence of other ions. A linear range of 10–100 µM and a detection limit of 0.64 nM were reported [135].

Qi et al. [136] reported a practical and sensitive aptamer-based colorimetric assay for Hg^2+^ by observing (visual and spectrum detection) changes in cationic gold nanoparticles (AuNPs). The observed changes were based on the affinity interaction of the nanoparticles with the mercury aptamer in the presence or absence of Hg^2+^. This investigation highlighted the progress that has been made with aptamer recognition for analysis and detection, as well as AuNP colorimetric analysis using aptamer recognition technology. Cationic AuNPs were used to distinguish the conformation formed by the affinity action of the aptamer and mercury (T-Hg-T) from the original conformation of the aptamer without mercury. This approach was taken to avoid any indirect responses that would have been induced by salt addition if the alternate anionic nanoparticles had been used. Their results showed that in the absence of Hg^2+^, the aptamer had its original single-stranded DNA conformation and would easily wrap around the surface of the AuNPs, causing them to agglomerate. Here, the resulting cationic AuNP solution was blue in appearance. Conversely, in the presence of Hg^2+^, due to high-affinity interactions with their aptamers, a solid T-Hg-T conformation was formed. The rigid aptamers were then unable to wrap onto the cationic AuNPs, causing the AuNPs to remain in their dispersed state, and the solution appeared red. Experimental results showed that Hg^2+^ concentration ranges from 8.2 × 10^−10^ to 6.2 × 10^−8^ M had high sensitivity correlation with an absorbance ratio of (A_650_/A_700_) for detecting Hg^2+^ with a limit of detection of 4.9 × 10^−11^ M. This aptamer–AuNP system was also selective for Hg^2+^ when applied to actual environmental samples. The advantage here is that once the aptamer of any polluting metal is used, then this presented method could be applied to detecting that heavy metal. Thus, a practical colorimetric analysis involving the simple synthesis of metal-specific probes was illustrated [136].

Bimetallic nanoparticles can also find application as colorimetric sensors. For example, Kheibarian and colleagues synthesized Cu@Ag core-shell NPs using the aqueous extract *Citrus paradisi* peel as a reducing and stabilizing agent [137]. They successfully used the system for the selective colorimetric detection of Hg^2+^ ions in an aqueous solution. Their reasoning for the copper core with a silver shell nanoparticle design was to improve stability and copper core functionality. Additionally, the flavonoids and polyphenols in the *Citrus paradisi* peel served as reducing and stabilizing agents in synthesizing the bimetallic nanoparticles. The coupling of these two metals forms bimetallic nanoparticles with improved surface plasmon resonance. Ultraviolet–visible (UV-Vis) characterization of the synthesized mixture showed a Cu@Ag NPs absorbance band at 411 nm, indicating that the Cu-NPs were coated with Ag-NPs; several other characterization techniques were also used to confirm the synthesis of the intended nanoparticle design. When Hg^2+^ ions were added to the Cu@Ag NP solution, the color of the solution changed from yellow to pink, with the absorbance band at 411 nm decreasing and a second absorption band appearing at 492 nm. Their sensitivity and selectivity analysis of the colorimetric sensor to other metals, including Ni^2+^, Cd^2+^, and Pb^2+^, showed that these metals did not interfere with the detection of Hg^2+^ ions. The UV-Vis absorbance of Cu@AP NPs with different concentrations of Hg^2+^ ions gave good absorbance ratio (A_492_/A_411_) correlations, with a detection limit of 5 × 10^−6^ M [137].

#### 3.1.6. Detection of Hg^2+^ and Pb^2+^ Ions

Chadha and coworkers reported the synthesis of a novel 2-thiazoline-2-thiol (TT) functionalized gold (Au-TT) nanosensor for the colorimetric detection of Hg(II) and Pb(II) ions in aqueous solutions [138]. Colloidal AuNPs were prepared and functionalized by adding different concentrations of TT to the nanoparticles. Observed color changes of the Au-TT nanoprobe colloidal solution ranged from bright red to purple in the presence of Hg(II) ions, to bright red to blue in the presence of Pb(II) ions. The absorption spectrum of the Au-TT sensor showed a bulk-like surface plasmon resonance (BL-SPR) band with a maximum at A_521_. There was a slight increase in the band absorbance when an Hg(II) solution was added to the nanosensor. However, when a Pb(II) solution was added to the probe, not only did the BL-SPR band decrease in intensity, but a new shoulder/red-shifted peak was also observed. Furthermore, the investigators gave characterization evidence to show that the observed color changes resulted from differences in the binding affinities of the metal ions towards the active binding sites of TT. The Au-TT nanosensor was also tested for its selectivity and specificity using various metal ions and actual samples from different water sources. The sensor was shown to be selective towards Hg(II) and Pb(II) ions, with high specificity towards Pb(II) ions in the presence of all other metal ions, at a limit of detection of approximately 100 ppb [138].

#### 3.1.7. Detection of Hg^2+^ and Cd^2+^ Ions

Wang et al. [139] developed a rapid and convenient method to detect Hg(II) and Cd(II) ions in water samples. They prepared a colorimetric sensor that utilized an aptamer made from a thymine (T) rich sequence (ssDNA (Hg)) to regulate the oxidase-mimicking activity of Mn_3_O_4_-NPs. In an acidic solution, the chromogenic substrate 3,3′,5,5′-tetramethylbenzidine (TMB) is oxidized by Mn_3_O_4_-NPs to give a yellow-colored solution. The aptamer served dual purposes. Firstly, the ssDNA (Hg) sequence was absorbed onto the surface of certain shaped Mn_3_O_4_-NPs, limiting their catalytic oxidation of TMB. This led to a color change of the solution from yellow to light green, and a smaller absorption peak was observed at 450 nm. Secondly, in the presence of Hg(II) and Cd(II) ions, the ssDNA (Hg) sequence would bind to these metals and no longer inhibit the oxidase-mimicking activity of the Mn_3_O_4_-NPs. The color of the sensing solution was then restored to yellow, with the increase in absorbance at 450 nm corresponding to the amounts of heavy metals present. The method was reported to be cost-effective, easy to use, and allowed Hg(II) and Cd(II) to be detected at concentrations as low as 20 µgL^−1^, with detection limits of 3.8 µgL^−1^ of Hg(II) and 2.4 µgL^−1^ of Cd(II), respectively. The investigators of this study believe that the method could be extended to detect other metals of interest by simply incorporating target-specific aptamers [139].

#### 3.1.8. Detection of Cd^2+^ and Ni^2+^ Ions

In a green synthesis method, Mohammadzadeh et al. [140] used a green walnut husk (GWH) extract for the synthesis of phenolic capping silver nanoparticles (PC-Ag NPs) to colorimetrically detect Cd^2+^ and Ni^2+^ ions in surface water and groundwater samples. In this investigation, the phenolic content of the Persian walnut (*Juglans regia* L.) extract was utilized as a reducing and stabilizing agent to synthesize Ag NPs. The PC-Ag NPs were prepared by adding GWH extract to an AgNO_3_ solution, in which the phenolic compounds in the extract were adsorbed on the surface of Ag^+^ ions and stabilized the formed nanoparticles. The LSPR absorbance band for the PC-Ag NPs was 445 nm. The synthesis of these nanosensors was confirmed when the solution turned from pale yellow to brownish yellow. The optical and chemical properties of the synthesized PC-Ag NPs were assessed using various characterization techniques. The colorimetric sensing mechanism was based on the reaction of the polyphenol functional groups (i.e., carboxyl and hydroxyl) of GWH on the surface of the nanosensors with Cd^2+^ and Ni^2+^ ions. In turn, this reaction led to the formation of chelate complexes with the metal ions, causing aggregation (with Cd^2+^ and Ni^2+^ ions) and sedimentation (with only Ni^2+^) of the nanoparticles. A visual color change from brownish yellow to pale grey was also seen when Cd^2+^ and Ni^2+^ ions were added to the nanosensors. Moreover, with increasing concentrations of Cd^2+^ and Ni^2+^ ions in the PC-Ag NP solutions, the LSPR absorbance band gradually decreased. Likewise, a gradual redshift was seen due to the aggregation of the PC-Ag NPs. The detection limit for both metal ions was 0.2 nM, and the linear range was between 0.05 and 100 µM. The sensor was found to have good selectivity and sensitivity at an optimized pH of 6 [140].

#### 3.1.9. Detection of Various Heavy Metal Ions

In a similar green synthesis investigation to Augen et al. [132] and Mohammadzadeh et al. [140], Sharma and coworkers [147] synthesized pectin-functionalized nanoparticles (P-AgNPs) using a microwave-assisted method. Likewise, in this study, pectin was used as a reducing and stabilizing agent during the synthesis of the AgNPs. The pectin-functionalized nanoparticles were evaluated by various characterization techniques, and they had a maximum absorbance of 409 nm. Subsequently, the synthesized P-AgNPs were utilized for the colorimetric detection of a broad range of metal ions (Fe(II), Mn(II), Cr(III), Cr(VI), and As(V)) in aqueous solutions. When an aqueous colloidal P-AgNP solution was added to water samples containing Fe(II) and Mn(II) ions, the solution’s color changed from light yellow to black and dark brown, respectively. The solution’s color intensity depended on the concentration of these metal ions in the water. At low concentrations of Fe(II), the LSPR peak of the P-AgNPs shifted to 440 nm, whereas at high concentrations of this metal ion, new bands were formed at 375 and 475 nm. The researchers found that adding Mn(II) ions changed the LSPR band of P-AgNPs while slightly increasing the absorbance. However, high concentrations of Mn(II) ions led to distortion of the absorption peak due to agglomerate formation. No significant color change was observed when water samples containing a low concentration of Cr(III) ions were mixed with the colloidal P-AgNP solution. Conversely, at medium-to-high concentrations (40–90 µM) of Cr(III) ions, the solution’s color changed from light yellow gradually to reddish brown and finally to intense brown at 100 µM. Likewise, these visual color changes with a Cr(III) concentration increase correlated to an absorption increase of the LSPR peak. The authors also observed a Cr(III) concentration of 40–100 µM, a downward peak shift seen at 400 nm, and, between 70 and 100 µM, an additional peak at 479 nm. On the other hand, adding an aqueous Cr(VI) ion solution to a colloidal P-AgNP solution led to a slight blue shift in the LSPR peak, which became higher as the Cr(VI) concentration increased. The P-AgNP solution changed from light yellow to pale yellow. It was found that the color intensity was not concentration dependent. The authors suggested this detection pattern could be helpful for the preliminary screening of Cr(III) and Cr(VI) ions. When As(V) ions at low (2–25 µM), medium (35–50 µM), and high (>50 µM) concentrations were mixed with P-AgNPs, a color change from light yellow to red, brown, and light yellow (no color change) was observed, respectively. The As(V) caused in the presence of P-AgNPs a blue shift of only 1 nm. At low and medium concentrations of As(V) ions, there was a direct proportional relationship with the absorbance, indicating a complex formation between As(V) ions and P-AgNPs. However, at high concentrations of As(V) ions, there was an inversely proportional relationship with the absorbance. In this study, the researchers found that the variations in color and LSPR absorption fingerprint of P-AgNPs were due to the formation of nanoparticle aggregates with the added metal ions. Results show that the pectin functional groups (e.g., carboxyl and hydroxyl) on the nanoparticles’ surfaces formed a complex with the heavy metal ions through metal–ligand interaction. This caused the dispersed P-AgNPs to become aggregated, causing the nanoparticles to be closer to each other and ultimately resulting in a change in peak position and color change. The data from this investigation indicate the potential use of these P-AgNP sensors as a screening and detection method for various metal ions. This colorimetric detection method was straightforward, fast, and sensitive [147].

### 3.2. Colorimetric Nanosensors for Detecting Heavy Metal Ions in Biological Samples

Colorimetric techniques have become popular recently because of on-the-spot detection, sometimes with the naked eye and without sophisticated instrumentation. Ullah et al. [141] synthesized highly sensitive, acyclovir-stabilized silver nanoparticles (AC-AgNPs) that were selective for sensing Hg^2+^ ions and found application in detecting Hg^2+^ ions spiked in human blood plasma samples. The AC-AgNPs were synthesized using a chemical reduction method, where a color change from colorless to yellow indicated successful synthesis of the AC-AgNPs. The role of the acyclovir was to stabilize Ag^+^ and reduce it by donating an electron pair. The presence of functional groups on the stabilizer also served to enhance the binding of Hg^2+^ onto the AgNPs surface. The synthesized AC-AgNPs were initially characterized using UV-Vis spectroscopy at a wavelength of 404 nm, along with other characterization techniques. The AC-AgNPs were small (44.1 nm) with uniform morphology and size distribution and had a zeta potential of −17.4 mV. The AC-AgNPs, when mixed with Hg^2+^ ions, showed variations in surface plasmon resonance, and gave an absorption spectrum with hypochromic and hypsochromic shifts and observable color change of the sensor solution from yellow to greyish. Data from their experiments suggested a 1:2 binding stoichiometry between AC-AgNPs and Hg^2+^ ions, with a detection limit of 0.00035 mM. Moreover, the nanosensor was selective for Hg^2+^ ions even in the presence of interfering metal ions (Ca^2+^, Ba^2+^, NH_4_^+^, Cu^2+^, Pb^2+^, Co^2+^, Cr^2+^, Al^3+^, Pb^2+^, Fe^2+^, Ni^2+^) in various samples. Thus, the results of this study showed the potential of the designed colorimetric nanosensor for the selective and easy detection of Hg^2+^ in different media [141].

In a related study, Liu et al. [142] developed a colorimetric method that was highly selective and sensitive to Hg^2+^ ions and successfully used it to detect mercury in human blood. They fabricated covalent organic frameworks (COFs) and grew noble silver nanoparticles (Ag-NPs) onto the COF surfaces in situ, via a one-step chemical reduction method, to yield COF-Ag nanozymes. Characterization with a transmission electron microscope (TEM) showed the COF-Ag nanozymes formed a monodispersed hierarchical flower-like structure with uniform nanoparticles on the surface of the pores of the COFs. The COF-nanozymes possessed oxidase-like catalytic activity that was enhanced in the presence of Hg^2+^ ions, forming Ag-Hg alloys. This oxidase-like catalytic activity was observed with the colorimetric substrate 3,3′,5,5′-tetramethylbenzidine (TMB): in the presence of Hg^2+^ ions the oxidase activity of the COF-Ag nanozyme with TMD resulted in a blue colored solution, while in the absence of Hg^2+^ ions the solution remained colorless. The COF-Ag nanozymes with and without mercury were characterized by UV-Vis spectroscopy, where COFs were taken as a control. Observations showed that adding Hg^2+^ ions enhanced the absorbance of COF-Ag nanozymes, which was postulated to be due to the scattering effect of the formed Ag-Hg alloys. Detection of different concentrations of Hg^2+^ ions spiked in blood samples gave favorable recoveries and proved the reliability of the colorimetric method for determining Hg^2+^ ions in blood samples; a linear concentration range from 0.050 to 10.0 µM and a limit of detection of approximately 3.7 nM was reported for this colorimetric method. It was also shown that other measured ions (example Fe^3+^, Cu^2+^, Ca^2+^, Cd^2+^, Zn^2+^, Pb^2+^, Cr^3+^, I^−^, SrO_3_^2−^, S^2−^) had negligible influence on the catalytic activities of COF-Ag nanozymes, when compared to Hg^2+^, thus confirming the high detection selectivity of the reported colorimetric method for Hg^2+^ ions [142].

A novel process towards the detection of Arsenite (As(III) in tissue samples (aka viscera/internal organs) using polyethylene glycol capped gold nanoparticle (PEG-AuNPS) nanocomposites was reported by Shalvi et al. [143]. In the study, the PEG-AuNPs were prepared and characterized using several characterization techniques. It was shown that electrostatic interaction caused the aggregation of As III ions on binding with the nanocomposites, which resulted in a color change from wine-red to blue. An optical hand-held device was fabricated in-house and used to quantify trace amounts of As III ions in the samples based on absorbance at 612/521 nm. The development and integration of the hand-held device with the nanocomposites facilitated the on-the-spot quantification of As III ions in tissue samples. The sensing capability of the hand-held device using the PEG-AuNPs showed good linearity (0.1–10 ppm) and correlation when compared with standard methods. The developed PEG-AuNPs were reported to be sensitive and selective in detecting As III ions in the presence of interfering components (for example Ca^2+^, Cd^2+^, Cu^2+^, Na^+^, Ni^2+^, Al^3+^, Hg^2+^, Mn^2+^, Mg^2+^, and Zn^2+^), with their hand-held device having a detection limit in tissue samples of 2.9 ppm [143].

In a separate investigation by Zhang et al. [144], AuNPs functionalized with cysteamine aptamer were used to detect As(III) ions in artificial urine. They developed a dual-mode (dispersion and aggregation) colorimetric method for the determination of As(III) ions that was based on specific recognition and electrostatic interaction between As III ions, As III aptamer (As III-apt), and positively charged gold nanoparticles (+AuNPs). The wine-red colored +AuNPs were prepared and characterized. Following that, the negatively charged AsIII-apt solution was prepared and used to functionalize the +AuNPs. The aptamer solution served to regulate the aggregation and dispersion of the +AuNPs, via electrostatic interactions between the nanoparticles and the aptamer. The color of the +AuNP solution changed from red to blue to red as the concentration of the AsIII-apt increased. The absorbance of the reaction solutions was measured at 680 nm (A_680_), which calculated the relative amounts of aggregated +AuNPs, as well as at 526 nm (A_526_), which determined the relative amounts of dispersed +AuNPs. Thus, the ratio of A_680_/A_526_ represented the ratio of aggregated to dispersed +AuNPs. Final concentrations of 8 nM of As III-apt (for the dispersed mode) and 15 nM (for the aggregated mode) were used to determine As(III) ions in natural samples, as well as artificial urine. Thus, in this biosensor, the aggregation of +AuNPs resulted when the aptamer concentration was low (8 nM). Then, as As(III) ions were introduced, a complex formed between the As III-apt and As III ions. The depletion of the aptamer caused the +AuNPs to continue to be dispersed in the solution. Conversely, with increased concentration of As III-apt (8–15 nM), electrostatic interactions caused the +AuNPs to remain dispersed in the detection system. Then, as As III ions were introduced, the As(III) ions would complex with the AS III aptamer, depleting the amount of aptamer adsorbed onto the surface of the +AuNPs, causing them to aggregate. In this study, the LOD for detecting As(III) ions in urine using the biosensor in aggregation mode was reported to be 0.41 ppb with a linear range of 2–40 ppb (R^2^ = 0.996). In addition, the authors reported that their use of +AuNPs and aptamer proved to be advantageous, as the detection process and detection time were simplified and shortened, when compared to their previous methods which used negatively charged AuNPs, cationic polymer, or salts and aptamer [144].

### 3.3. Detection of Heavy Metal Ions in Consumable Products

The colorimetric determination of heavy metals using nanosensors has also found application in the food industry. The metal ion cadmium is a highly toxic, carcinogenic contaminant that adversely affects human health [148]. Non-industrial exposure may arise from cigarette smoke and food (via soil and water contamination). Milk and dairy products can become contaminated with cadmium ions from either adulteration or dilution with water [149]. Therefore, an accurate and selective method for detecting and monitoring this element is essential. Sonia and Raman [145] developed an *L*-Cysteine modified gold nanoparticle (AuNP)-based colorimetric assay technique for detecting the toxic metal ion of cadmium in milk samples. They described their technique as a simple and low-cost alternative compared to other spectroscopy-based methods. The authors reported that synthesized colloidal AuNPs possessed strong SPR absorptions with high extinction coefficients in the visible range; they believe these properties depend on the shapes and sizes of the AuNPs, the dielectric constant of their surrounding aqueous media, and interactions with neighboring particles. In their study, 24 nm spherical AuNPs were prepared from reducing gold (III) chloride trihydrate with sodium tri-citrate. The AuNPs were later functionalized with *L*-cysteine. Their synthesized *L*-cysteine AuNPs were dark red in color, spherical when viewed under a transmission electron microscope (TEM) and had the strong ability to form aggregates with toxic metal ions, leading to a color change. The *L*-cysteine acted as a target-specific ligand, facilitating binding to target metal ions. When metal ions (Cd^2+^) were introduced, they would bind through the ligands of multiple AuNPs, inducing nanoparticle aggregation, which resulted in a new ultraviolet–visible band at 520 nm and an observable color change of the red AuNPs into a deep blue [145].

Vonnie et al. [146] also developed a sensitive, simple, uncomplicated, and environmentally friendly colorimetric detection method using a film of tapioca starch and gold nanoparticles (TS-AuNPs), which was selective for cadmium ions (Cd^2+^) in fish. Significant absorbance was observed at a wavelength of 620 nm when a cadmium solution was added to the sensor. Moreover, the TS-AuNPs showed increased response to Cd^2+^ when compared to other metal ions (Hg^2+^, Ni^2+^, Fe^2+^, Pb^2+^ and Cu^2+^). The tapioca starch (TS) thin film was used as the colorimetric reagent carrier, which allowed for on-site detection. The AuNP aggregates were produced from the reaction of chloroauric acid and sodium citrate. The subsequent formation of negatively charged AuNPs on the surface of the TS thin film was stabilized by citrate ions, which allowed them to remain dispersed in the solution. The detection process was based on the affinity level of the heavy metal ions (Cd^2+^) and their ability to exert an attractive force on the negative surface of the Au-NPs. The detection mechanism was based on aggregate formation between the AuNPs and cadmium, which resulted in a color change of the solution from red to purplish grey. The reported UV-Vis curves of the colorimetric response of the AuNPs and cadmium showed linearity in the cadmium concentration range from 6 mmol·L^−1^ to 12 mmol·L^−1^ (R^2^ = 0.9935) and an LOD of 13.1 mmol·L^−1^. Furthermore, the edible parts of seven deep-sea fish species were tested (*Thannus obesus*, *Scomberomorus commerson*, *Euthynnus affinis*, *Nemipterus furcosus*, *Selar crumenophthalmus*, *Pracanthus lovetii*, and *Megalaspis cordyla*). The results showed all of them to be contaminated with Cd^2+^ at levels that were reported to be higher than Cd^2+^ permissible values [146].

## 4. Conclusions and Future Directions

This review highlighted the innovations and advancements in electrochemical and colorimetric nanosensors for heavy metal ions in a variety of samples of ecological, environmental, biological, and consumable interest. The quality and high volume of published articles in high-impact journals demonstrate a continued interest and the critical need to develop an analytical protocol for rapidly detecting heavy metal ions to prevent environmental contamination and heavy metal ion poisoning to ensure public health safety. Notable achievements and progress have been made in developing sensors capable of accurate and reproducible heavy metal ion detections at trace and ultra-trace concentrations. Nonetheless, sensitivity, selectivity, specificity, and interference remain challenging for several available sensors. Considerable efforts will be devoted to developing improved electrochemical and colorimetric nanosensors with better selectivity, sensitivity, and specificity to facilitate reliable and expanded heavy metal ion detections. Specifically, efforts will be dedicated to the instrumental design and development of more portable electrochemical sensors based on graphene, carbon nanotubes, nanostructures, carbon dots, nanomaterials, and metal–organic frame sensors to promote selective and specific heavy metal ion detections. The development of portable colorimetric sensors that will promote fast screening and visual detection of heavy metal ions will continue to be an active research area in the coming years. Improved technology and smartphone access will enable the wide application and development of smartphone-based sensors that will facilitate rapid, in situ, and on-site detection of heavy metal ions. Several low-cost and disposable paper-based sensors will be developed that will facilitate in situ and field detection of heavy metal ions. Microfluidic and microchip sensors will generate more research interest to promote rapid arrays and simultaneous heavy metal ion detections. Using functionalized gold nanoparticles for fiber-optics surface plasmon resonance for heavy metal ion sensing will attract greater interest. In addition, more articles will report on using automated and robotic sensors for heavy metal ion detections.

## Figures and Tables

**Table 3:** Recent advances in colorimetric (UV-Vis) nanosensors for heavy metal ion detection.

Heavy Metal Target	Nanosensor Material	Observed/Detected Color Change (Absorbance Wavelength of Interest)	Sample Type Examined	LOD	Linear Range	Ref.
Environmental Samples						
Cu^2+^	Thiazolylazopyrimidine-functionalized TiO_2_ nanosensor (TiO_2_-TAP)	Yellow to red (A_536_)	Water	2.51 nM	0.01–12.5 µM	[130]
Cr^3+^ and Cu^2+^	Multi-functional iodide-assisted silver nanoplates	Deep yellow to purple (A_390_/A_520_) for Cr^3+^; Deep yellow to colorless (A_390_) for Cu^2+^	Environmental water samples	8.0 nM for Cr^3+^; 0.27 µM for Cu^2+^	25–400 nM for Cr^3+^; 0.3–10 µM for Cu^2+^	[131]
Fe^3+^, Cu^2+^, and Cr^6+^	Ag@AgCl NPs	Dark brown to light brown for Fe^3+^; Dark brown to white for Cu^2+^; Dark brown to orange for Cr^6+^ (A_400–500_)	Environmental water samples	1.69 ppb for Fe^3+^; 3.18 ppb for Cu^2+^; 5.05 ppb for Cr^6+^	0–100 ppb	[132]
Pb^2+^	G-AuNPs	Claret-red to gray (A_530_)	Environmental water samples	1.07 µM	10–80 µM	[133]
Hg^2+^	L-Cysteine functionalized graphene oxide nanoarchitectonics CGO	Darker blue color of TMB oxidation products (A_652_)	Water	7.6 µgL^−1^	0–200 µgL^−1^	[134]
Hg^2+^	Graphene oxide stabilized AgNPs	Yellow to colorless (A_400_)	Environmental water samples	0.64 nM	10–100 µM	[135]
Hg^2+^	Aptamer-modified cationic AuNPs	Blue to red (A_560_/A_700_)	Environmental water samples	4.9 × 10^−11^ M	8.2 × 10^−10^ M~6.2 × 10^−8^ M	[136]
Hg^2+^	Cu@Ag NPs, stabilized with *Citrus paradisi* peel	Yellow to pink (A_492_/A_411_)	Aqueous solutions	5 × 10^−6^ M	Not reported	[137]
Hg^2+^ and Pb^2+^	2-thiazoline-2-thiol functionalized AuNPs	Bright red to purple for (Hg^2+^); bright red to blue for (Pb^2+^) (A_521_)	Water samples	~100 ppb	0.1–10 µM	[138]
Hg^2+^ and Cd^2+^	ssDNA (Hg) functionalized Mn_3_O_4_NPs	Light green-yellow (A_450_)	Water samples	3.8 µgL^−1^ for Hg^2+^ and 2.4 µgL^−1^ for Cd^2+^	Not reported	[139]
Cd^2+^ and Ni^2+^	PC-Ag NPs	Brownish-yellow to pale yellow (A_445_)	Environmental water samples	0.2 nM	0.05–100 µM	[140]
Biological Samples						
Hg^2+^	Acyclovir stabilized, silver nanoparticles AC-AgNPs	Yellow to greyish (A_404_)	Human blood plasma	0.00035 mM	Not reported	[141]
Hg^2+^	Silver nanoparticles on covalent organic frameworks COF-Ag nanozymes	Dark blue color of TMB oxidation products (A_652_)	Human blood	3.7 nM	0.050–10 µM	[142]
As^3+^	Polyethylene glycol-capped gold nanoparticles (PEG-AuNPs)	Wine red to blue (A_612_/A_521_)	Human tissues (viscera)	2.9 ppm	0.1–10 ppm	[143]
As^3+^	As^3+^ aptamer functionalized positively charged gold nanoparticle. As^3+^ -apt- +AuNPs	Blue to red (A_680_/A_526_)	Urine	0.41 ppb	2–40 ppb	[144]
Consumables						
Cd^2+^	L-Cysteine modified gold nanoparticles AuNPs	Red to blue (A_520_)	Milk	Not reported	Not reported	[145]
Cd^2+^	Film of Tapioca starch and gold nanoparticles Ts-AuNPs	Red-purplish to grey (A_620_)	Fish	13.1 mmol·L^−1^	6–12 mmol·L^−1^	[146]

## Data Availability

Data are contained within the article.
